# The Effectiveness of Clear Aligners Versus Fixed Aligners in Malocclusion Patients Undergoing Orthodontic Treatment: A Systematic Review and Meta-Analysis

**DOI:** 10.7759/cureus.96986

**Published:** 2025-11-16

**Authors:** Abdulaziz Owayed, Dhari M Alshammari, Sayed A Alsaleh, Lulwa Al Terkait, Abdulwahab N Alenezi, Asmaa M Alajmi, Abdulaziz H Alajmi, Mohamed Bourkhan, Yousef Alajmi, Ahmad S Shanat, Ahmed Abdelaziz

**Affiliations:** 1 Department of Dentistry, Al-Amiri Hospital Dental Center, Kuwait City, KWT; 2 Department of Dentistry, Salwa Specialized Center, Kuwait City, KWT; 3 Department of Dentistry, West Hawally Polyclinic, Hawally, KWT; 4 Department of Dentistry, School of Medical Sciences University of Leeds, Leeds, GBR; 5 Department of Dentistry, Saad Al-Abdullah Block 5 Polyclinic, Saad Al-Abdullah City, KWT; 6 Department of Dentistry, Jaber Al-Ali Polyclinic, Al Farwaniyah, KWT; 7 Department of Dentistry, Al-Omariyah Health Care Center, Al Farwaniyah, KWT; 8 Department of Dentistry, Oyoun Polyclinic, Al-Jahra, KWT; 9 Department of Dentistry, Al Qarain Health Center, Fnaitees, KWT; 10 Department of Biostatistics, Faculty of Medicine, Al-Azhar University, Cairo, EGY

**Keywords:** clear aligners, fixed aligners, malocclusion of teeth, oral health impact profile (ohip-14), systematic review and meta analysis

## Abstract

Malocclusion is one of the most significant dental issues that interferes with daily life activities. However, direct comparison between orthodontic treatment with clear aligners (CAs) and fixed aligners (FAs) is not yet established. We aimed to assess the impact of CA versus FA in patients with malocclusion. We searched PubMed, Scopus, Web of Science, and Cochrane Central Register of Controlled Trials (CENTRAL) from inception until October 2025 to identify randomized controlled trials (RCTs) comparing the impact of CA versus FA in malocclusion patients. The primary outcome of interest was the mean change of quality-of-health measures using the Oral Health Impact Profile-14 (OHIP-14) survey, while secondary outcomes were mean change in pain levels and in the objective grading system (OGS). Continuous outcomes were pooled as standardized mean difference (SMD) with its 95% confidence intervals (CIs) in a random-effects model. Stata/MP (StataCorp LLC., College Station, Texas, United States) was used to analyse all outcomes. We included seven RCTs comprising 402 patients in the analysis. Patients treated with CA had better OGS scores in buccolingual inclination and marginal ridges domains compared to those treated with FA (SMD= 0.74, 95%CI: 0.28-1.2, p <0.001, and 0.42, 95%CI: 0.16-0.68, p <0.001). However, there were no significant differences in other studied outcomes. Malocclusion patients treated with CA had a better clinical profile compared to those treated with FAs. Further long-term RCTs with standard malocclusion types are needed.

## Introduction and background

Malocclusion is a common dental condition characterized by misalignment of teeth. It can impair mastication, speech, oral hygiene, and physiological well-being, causing psychosocial distress. Orthodontic treatment aims to correct these deviations, improve occlusal function, and patient quality of life (QoL) [1]. 

For decades, fixed aligners (FAs) have been the gold standard of care for orthodontic treatment for dental malocclusion, offering precise control of tooth movement and providing long-term effectiveness. However, their metallic appearance, food retention, mucosal irritation, and oral hygiene challenges were associated with high rates of discomfort among the patients, leading to decreased QoL during the treatment period [2]. 

Over the last years, clear aligners (CAs), especially clear thermoplastic systems such as Invisalign® (Align Technology, Inc., Tempe, Arizona, United States), have gained increased popularity as an alternative to FAs. OAs are associated with better oral hygiene maintenance, reduced plaque accumulation, and improved patients' QoL and satisfaction compared to the traditional braces [3]. 

Although aligners can be comparable to FAs in treatment effectiveness, patient experience, and decreased side effects, they remain a subject of debate. Some studies report that aligners offer shorter treatment duration in mild to moderate cases, while others suggest that FAs have better control torque, occlusal contacts, and complex tooth movements [4,5]. A systematic review found no evidence that treatment duration differs between CAs and FAs in mild cases [5]. Another meta-analysis reported that aligners showed no significant difference from FAs in Little’s index and peer assessment rating (PAR) score, but lower plaque index, gingival index, and patient satisfaction [4,5]. 

Given the heterogeneous reported data, this systematic review and meta-analysis aims to comprehensively compare the effectiveness and patient-reported outcomes, such as pain and comfort, of CAs and FAs. 

## Review

Methods 

This systematic review and meta-analysis were conducted according to the predefined criteria of the Preferred Reporting Items for Systematic Reviews and Meta-Analysis (PRISMA) [6] and followed the guidelines proposed by the Cochrane Handbook of Systematic Review of Interventions [7]. 

Literature Search and Screening

An electronic search was conducted on PubMed, Scopus, Web of Science, and Cochrane Central Register of Controlled Trials (CENTRAL) databases from inception to October 2025 to identify all randomized controlled trials (RCTs) using the following key search terms: ((orthodontic aligner OR clear aligner OR Invisalign OR removable appliance) AND (fixed appliance OR conventional appliance OR brace OR bracket) AND ( malocclusion OR dental crowding OR misaligned teeth OR crooked teeth)). Detailed search strategy according to each specific database is shown in Table 1. In addition, the reference sections of all retrieved citations were searched for potential RCTs to ensure that we screened all potential studies. 

**Table 1:** Detailed search strategy for each database CENTRAL: Cochrane Central Register of Controlled Trials

Database	Search Terms	Search Field, Applied Filters	Search Date	Search Results
PubMed	((orthodontic aligner* OR clear aligner* OR "invisalign" OR removable appliance* ) AND ("fixed appliance*" OR "conventional appliance*" OR brace* OR bracket* ) AND ( malocclusion OR "dental crowding" OR "misaligned teeth" OR "crooked teeth" ))	All Field, English, Humans	October 16, 2025	294
CENTRAL	((orthodontic aligner* OR clear aligner* OR "invisalign" OR removable appliance* ) AND ("fixed appliance*" OR "conventional appliance*" OR brace* OR bracket* ) AND ( malocclusion OR "dental crowding" OR "misaligned teeth" OR "crooked teeth" ))	Title, Abstract, Keywords, English	October 16, 2025	116
Web of Science	((orthodontic aligner* OR clear aligner* OR "invisalign" OR removable appliance* ) AND ("fixed appliance*" OR "conventional appliance*" OR brace* OR bracket* ) AND ( malocclusion OR "dental crowding" OR "misaligned teeth" OR "crooked teeth" ))	All Field, English	October 16, 2025	234
Scopus	((orthodontic aligner* OR clear aligner* OR "invisalign" OR removable appliance* ) AND ("fixed appliance*" OR "conventional appliance*" OR brace* OR bracket* ) AND ( malocclusion OR "dental crowding" OR "misaligned teeth" OR "crooked teeth" ))	Title, Abstract, Keywords, English, Human	October 16, 2025	137

All screening steps were performed in a double-blind fashion. First, titles and abstracts were screened, followed by full-text screening for all eligible studies that met our predefined inclusion criteria. 

Eligibility Criteria and Outcomes

We included all RCTs that met the following predefined criteria: (i) population: patients with malocclusion, (ii) intervention: CAs as the main intervention, and (ii) control: FAs as the main control group. The studies had to assess our outcomes of interest in an intention-to-treat analysis. 

The primary outcome of interest was the mean change of QoL assessed using the Oral Health Impact Profile-14 (OHIP-14) [8]. The OHIP-14 survey consists of seven different domains and 14 related questions, each rated from 0 “never” to 4 “often”. Secondary outcomes of interest were the mean change of the objective grading system (OGS) score [9]. The OGS score includes eight categories of alignment, interproximal contacts, marginal ridges, occlusal contacts, buccolingual inclination, overjet, occlusal relations, and root angulations. Additionally, pain levels were evaluated on a post-treatment basis as per Cochrane standards for reporting post-treatment values [7].

Quality Assessment

Two reviewers assessed the risk of bias assessment using the Cochrane risk of bias assessment tool version 2 (ROB-2) [10]. The revised version ROB-2 tool assesses five main domains as follows: bias arising from the randomization process, bias arising from the deviation from the intended intervention, bias arising from missing outcome data, bias arising from the measurement of the outcome, and bias arising from the selection of the reported result. Each domain was judged as low risk, some concerns, and high risk of bias. Any disagreements between the authors were resolved via discussion with another author. 

Data Extraction and Statistical Analysis

We used a standardized Excel sheet (Microsoft Corporation, Redmond, Washington, United States) to extract all relevant data from the studies included. The extracted data were as follows: (i) baseline characteristics of the patients included age, sex, the complexity of the disease assessed by little's index [4], and severity of the disease assessed by PAR index [5], (ii) summary characteristics of the studies included including study design, inclusion and exclusion criteria, and the key findings of each study, (iii) risk of bias domains, and (iv) studied outcomes. 

Continuous outcomes were extracted as mean, standard deviations (SDs), and total number of patients, and were pooled as standardized mean difference (SMD) with its 95%CI using DerSimonian-Laird random effects model. The analysis was performed at one month of follow-up and at the end of treatment follow-up time. We assessed the heterogeneity using the Cochrane Q test, and the I2 measure was determined across all the studies. A significant heterogeneity among the studies was determined when a p-value was less than 0.05 and I2 ≥50%. Stata/MP release 19 (StataCorp LLC., College Station, Texas, United States) was used to perform all the statistical analyses using “meta esize” and meta forest plot” packages. 

Results 

Literature Search

**Figure 1:**
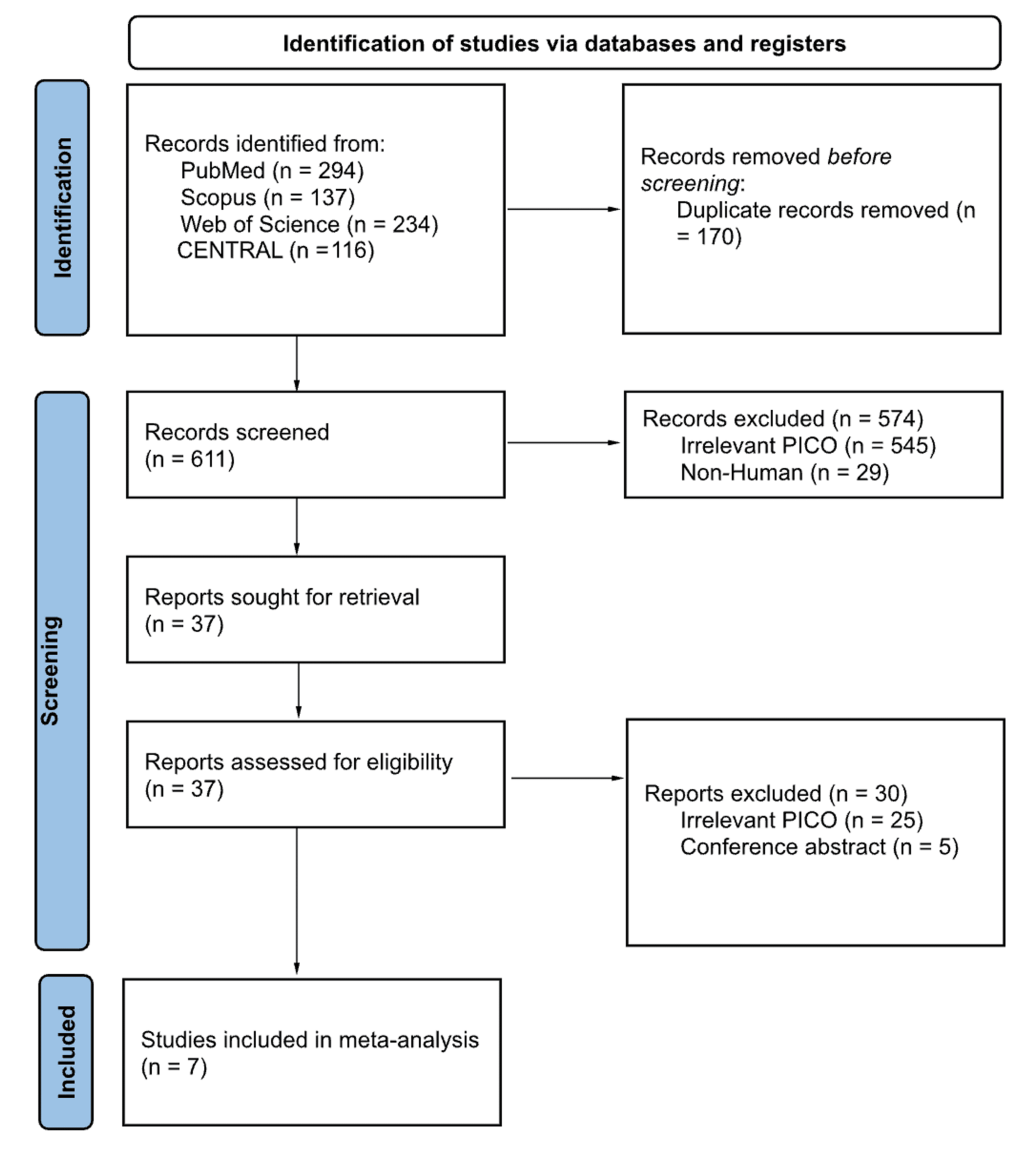
PRISMA flow chart PRISMA: Preferred Reporting Items for Systematic Reviews and Meta-Analysis; CENTRAL: Cochrane Central Register of Controlled Trials; PICO: Patient, Intervention, Comparison, and Outcome

A total of 611 citations were included in the titles and abstracts screening following duplicate removal. Of these, 37 were considered for full-text screening. Finally, we included seven RCTs in the overall systematic review [11-17], while six RCTs entered the analysis [11-14,16,17]. The detailed selection process is shown in the PRISMA diagram (Figure 1). 

Patients’ Characteristics and Risk of Bias Assessment

A total of seven RCTs comprising 402 patients were included in the final analysis. Of these, 201 (50%) patients were allocated to the CA group, while 201 (50%) were allocated to the FA group. Female patients were 217, comprising 53.9% of the total included patients. Additionally, the mean age of the included patients was 24.8 years. The detailed summary and baseline characteristics of the included studies and patients are shown in Tables 2, 3. 

**Table 2:** Summary characteristics of the included studies. OHIP-14: Oral Health Impact Profile-14; OGS: objective grading score

Study (Author, year)	Country	Study Design	Patients	Intervention	Control	Inclusion Criteria	Exclusion Criteria	Key Findings
﻿Borsato et al., 2025 [11]	Brazil	Parallel randomized clinical trial	38	Orthodontic treatment with clear aligners (Invisalign®)	Orthodontic treatment with conventional fixed metallic appliances	Male and female patients. Aged 13 to 35 years. Diagnosed with Angle’s Class I malocclusion. Moderate mandibular anterior crowding. No treatment involving tooth extraction.	Missing permanent teeth. Anterior or posterior open bite. Anterior or posterior crossbite. History of prior orthodontic treatment.	Clear aligners have less negative impact on patients' quality of life using OHIP-14 survey during the initial month of treatment compared to fixed appliances, but this difference disappears after six months.
﻿Jaber et al., 2023 [12]	Syria	Single-centre, parallel-group Randomized Controlled Trial	40	Orthodontic treatment with clear aligners	Orthodontic treatment with conventional vestibular fixed appliances	Adult patients aged 18–25 years. Angle's Class I malocclusion with severe crowding (>6 mm tooth size-arch length discrepancy). Treatment plan requiring extraction of all four first premolars. No congenitally missing or previously extracted teeth (except third molars).	Systemic diseases. History of trauma or surgery to the maxillofacial region. Psychological abnormalities. Previous orthodontic treatment. Known allergies to latex or plastic.	While no statistically significant difference was found in the overall OGS score, fixed appliances produced significantly better occlusal contacts and a clinically higher success rate, which should be considered when choosing a treatment modality for complex, extraction-based cases.
﻿de Leyva et al., 2023 [13]	Spain	Two-arm parallel Randomized Controlled Trial	28	Postsurgical orthodontic treatment with clear aligners (Invisalign®)	Postsurgical orthodontic treatment with traditional fixed appliances (Brackets)	Skeletal malocclusion requires combined surgical and orthodontic treatment. Treatment plan following a surgery-first approach (no pre-surgical orthodontics). No tooth extractions are required as part of the treatment.	Temporomandibular joint disorders or severe symptoms. Uncontrolled periodontal disease. Severe crowding requires extractions. Class II division 2 malocclusion with overbite or severely altered curves of Spee. Severe facial asymmetry.	For patients undergoing orthognathic surgery via a surgery-first approach, postsurgical orthodontic treatment with clear aligners (Invisalign) resulted in significantly better periodontal health and patient-reported quality of life outcomes compared to treatment with traditional fixed appliances, with no difference in overall treatment time.
Li et al., 2015 [14]	China	Multicenter Randomized Controlled Trial	152	Orthodontic treatment with the Invisalign® system	Orthodontic treatment with traditional fixed braces (Brackets)	Patients aged over 18 years. Treatment plan requiring tooth extraction. Class I malocclusion. Availability of high-quality pre- and post-treatment records (models, panoramic films). Signed informed consent.	Previous orthodontic treatment. Impacted teeth. Systemic diseases. Need for orthognathic surgery. Failure to comply with two-month follow-ups.	The study concluded that both Invisalign and fixed appliances were successful in treating Class I adult extraction cases, with a statistically significant improvement in the total OGS score for both groups from pre- to post-treatment.
Melo et al., 2021 [15]	Brazil	Parallel, randomized clinical trial	40	Orthodontic treatment with clear aligners (Invisalign®)	Orthodontic treatment with conventional fixed metallic appliances	Patients aged 13–35 years. Angle's Class I malocclusion. Moderate crowding. Treatment plan without tooth extraction.	Absence of permanent teeth. Anterior or posterior open bite or crossbite. Previous history of orthodontic treatment. Pre-existing speech impairments.	While a speech therapist objectively identified initial speech changes only in the aligner group, patients in both groups subjectively experienced temporary speech difficulties. Adaptation occurred for all patients within the first month of treatment.
﻿Tunca et al., 2024 [16]	Turkey	Single-centre, two-arm parallel-group randomized clinical trial	60	Clear aligner treatment (Invisalign®)	Conventional fixed orthodontic treatment	No prior orthodontic treatment. Angle Class I malocclusion. 4–6 mm arch length discrepancy in both dental arches. Permanent dentition Non-smokers, non-alcohol drinkers. No missing or impacted teeth, no plaque accumulation or gingival inflammation.	History of extraction fixed orthodontic treatment. Radiologically observed alveolar bone loss. Systemic disease. Use of drugs or analgesics during the survey period.	Clear aligners caused less initial pain and slightly better early quality of life, but no differences in anxiety or longer-term quality of life assessed by OHIP-14
Preston, 2017 [17]	United States	Randomized Controlled Trial	44	Clear aligner therapy (Invisalign®)	Fixed orthodontic appliances (Braces)	Adult Patients. Mild crowding. Class I malocclusion. Non-extraction treatment. No missing teeth (except third molars). Crowns and/or occlusal restorations were allowed if not broken.	Patients with broken restorations, missing teeth (except third molars), and requiring extraction treatment.	Both Invisalign and traditional braces produced similar final occlusal results, with no significant differences in posterior contact areas, marginal ridges, or buccolingual inclinations at the end of treatment or during the six-month retention period; however, the patient experience differed, as the Invisalign group reported significantly lower pain levels during the initial days of treatment, though treatment time was significantly longer for Invisalign compared to traditional braces.

**Table 3:** Baseline characteristics of cohorts in the included studies OA: ﻿orthodontic aligner; FA: ﻿fixed appliance; PAR: Peer Assessment Rating; NA: not assessed Little's Index (Little's Irregularity Index) assesses the complexity of the malocclusion [4], while the PAR index assesses the severity of a malocclusion (misaligned bite) [5].

Study ID	Group	Sample Size	Age (years), mean±SD	Sex		﻿Little’s Index	﻿PAR Index
				Male, n (%)	Female, n (%)		
﻿Borsato et al., 2025 [11]	OA	19	23.63±5.62	12 (75)	7 (25)	4.69 (1.35)	8.15 (4.89)
	FA	19	20.91±4.35	12 (75)	7 (25)	5.05 (1.91)	8.26 (3.57)
﻿Jaber et al., 2023 [12]	OA	20	21.3±2.37	6 (30)	14 (70)	NA	NA
	FA	20	21.46±2.53	8 (40)	12 (60)	NA	NA
﻿Leyva et al., 2023 [13]	OA	14	26.5±14.1	5 (35.7)	9 (64,3)	NA	NA
	FA	14	28.5±12.6	7 (50)	7 (50)	NA	NA
Li et al., 2015 [14]	OA	76	35.2±7.3	27 (35.5)	45 (59.2)	NA	NA
	FA	76	32.2±8.3	27 (35.5)	45 (59.2)	NA	NA
Melo et al., 2021 [15]	OA	20	23.6±5.65	12 (60)	8 (40)	4.69 (1.35)	7.7 (4.66)
	FA	20	20.56±5.51	13 (65)	7 (35)	4.99 (1.88)	7.5 (3.18)
﻿Tunca et al., 2024 [16]	OA	30	23.65±6.58	15 (50)	15 (50)	NA	NA
	FA	30	21.3±3.37	15 (50)	15 (50)	NA	NA
Preston, 2017 [17]	OA	22	27.8±NA	10 (47.6)	11 (52.4)	NA	NA
	FA	22	25.4±NA	7 (31.8)	15 (68.2)	NA	NA

**Figure 2:**
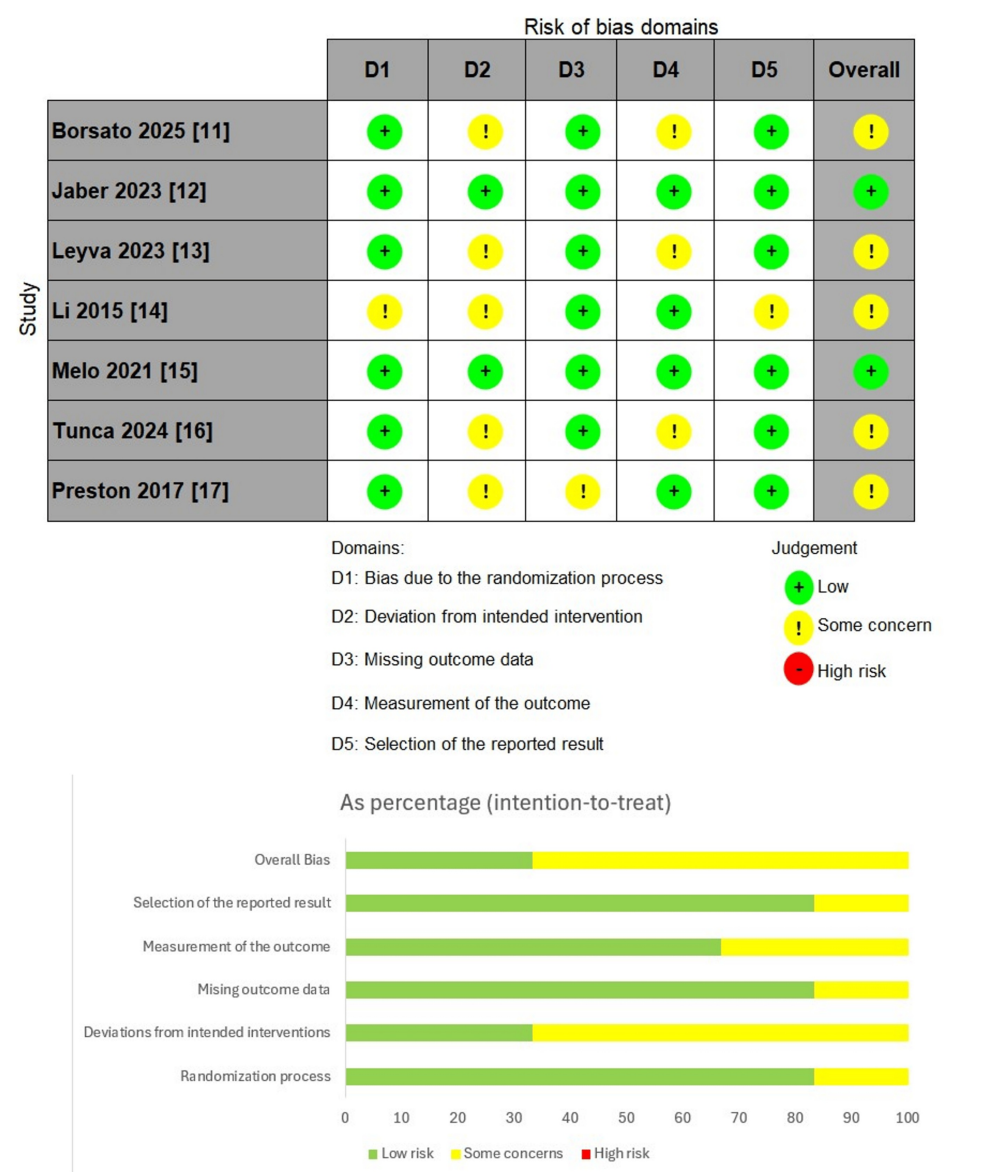
Risk of bias (ROB-2) assessment of the included studies References: [11-17]

We assessed the quality of the included RCTs using the ROB-2 tool. Five studies had overall some concerns, mainly due to deviations from intended interventions and measurement of the outcome domains, while only two studies had an overall low risk of bias (Figure 2). 

Outcomes 

**Figure 3:**
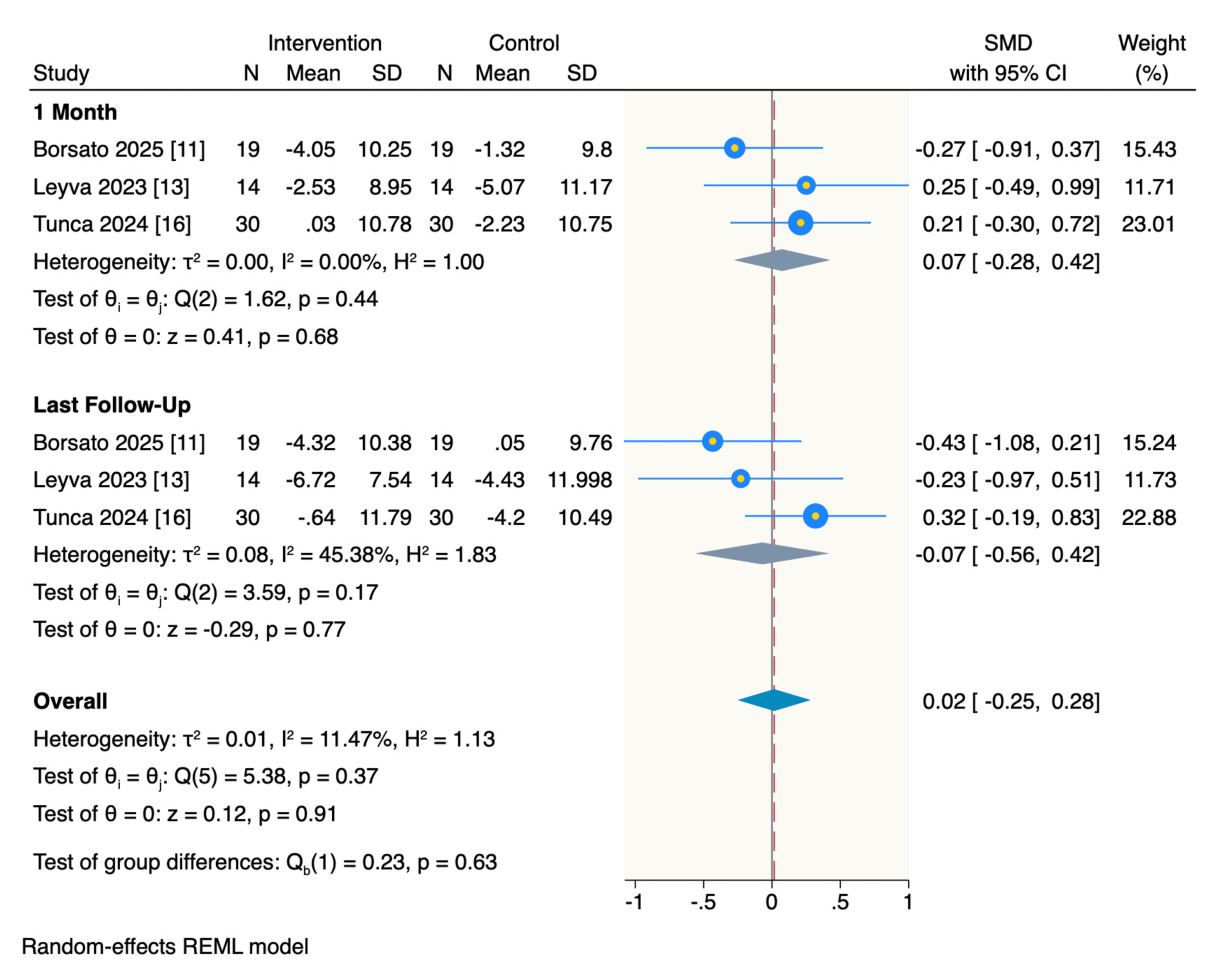
Random-effect model of the mean change of OHIP-14 score OHIP-14: Oral Health Impact Profile-14 [8] References: [11,13,16]

Primary Outcome (OHIP-14): There was no significant difference between the overall OHIP-14 survey scores between patients treated with CA or those treated with FA at one month of follow-up (SMD= 0.07, 95%CI: -0.28 to 0.42, p = 0.68; I2= 0.00%), or by last follow-up time which was the end of treatment point (SMD= -0.07, 95%CI: -0.56 to 0.42, p = 0.77; I2= 45.38%). The overall score at both follow-up points collectively was not significant either (SMD= 0.02, 95%CI: -0.25 to 0.28, p = 0.91; I2= 11.47%). There were no significant interactions between the two follow-up times (p = 0.63) (Figure 3). 

**Figure 4:**
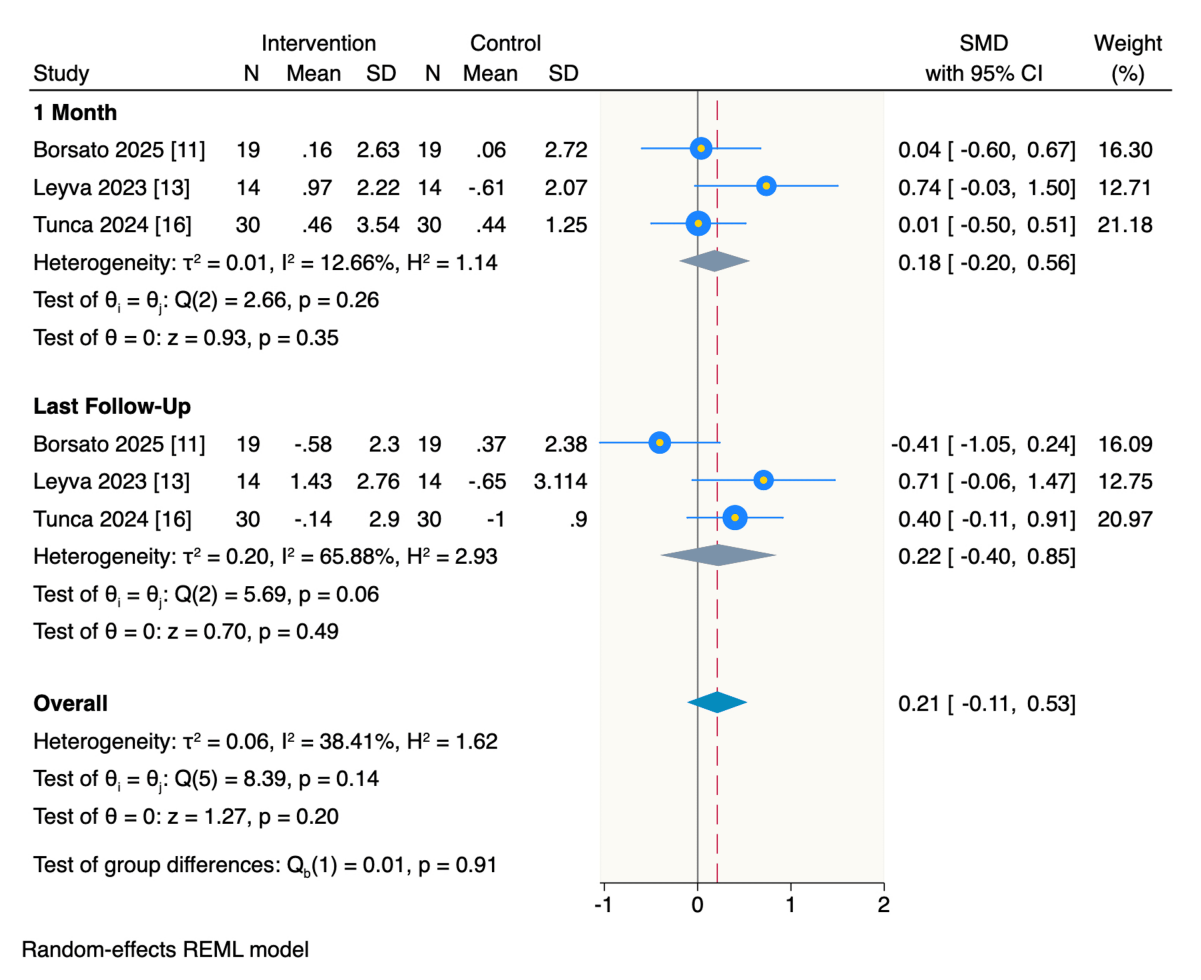
Random-effect model of the mean change of pain levels. The analysis of the pain levels was based on the Cochrane Handbook criteria for analysing post-treatment values [7] References: [11,13,16]

Secondary outcomes: Additionally, there was no significant difference between patients treated with CA and those treated with FA regarding their pain levels at one month of follow-up (SMD= 0.18, 95%CI: -0.2 to 0.56, p = 0.35; I2= 12.66%), or by last follow-up time which was the end of treatment point (SMD= 0.22, 95%CI: -0.4 to 0.85, p = 0.49; I2= 65.88%). There were no significant interactions between the two follow-up times (p = 0.91) (Figure 4). 

**Figure 5:**
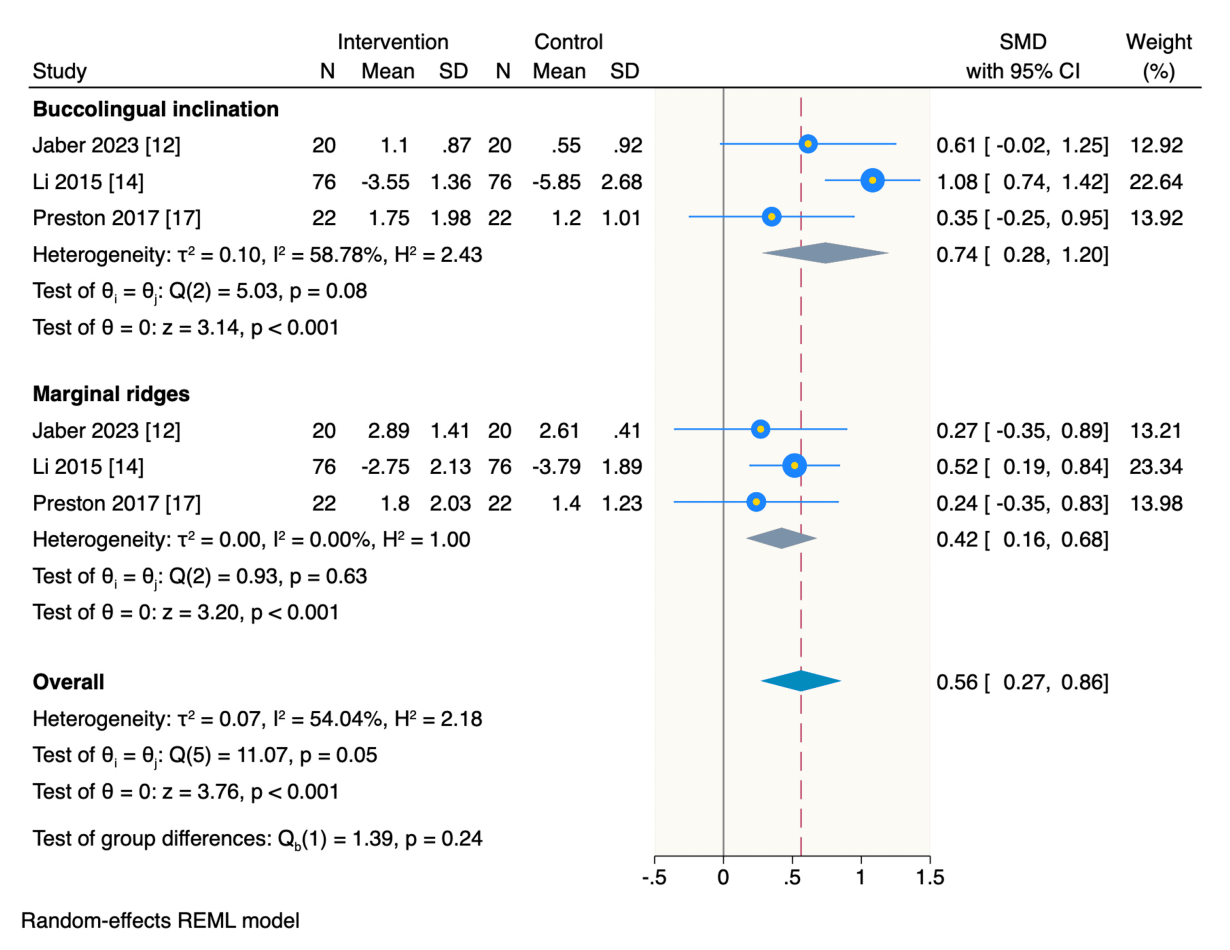
Random-effect model of the mean change of OGS scores of buccolingual inclination and marginal rides domains. OGS: objective grading score [9] References: [12,14,17]

On the other hand, patients treated with CA had significantly better OGS scores compared to those treated with FA in the buccolingual inclination domain (SMD= 0.74, 95% CI: 0.28 to 1.2, p <0.001; I2= 58.78%), and marginal ridges domain (SMD= 0.42, 95%CI: 0.16 to 0.68 p <0.001; I2= 0.00%) (Figure 5).

**Figure 6:**
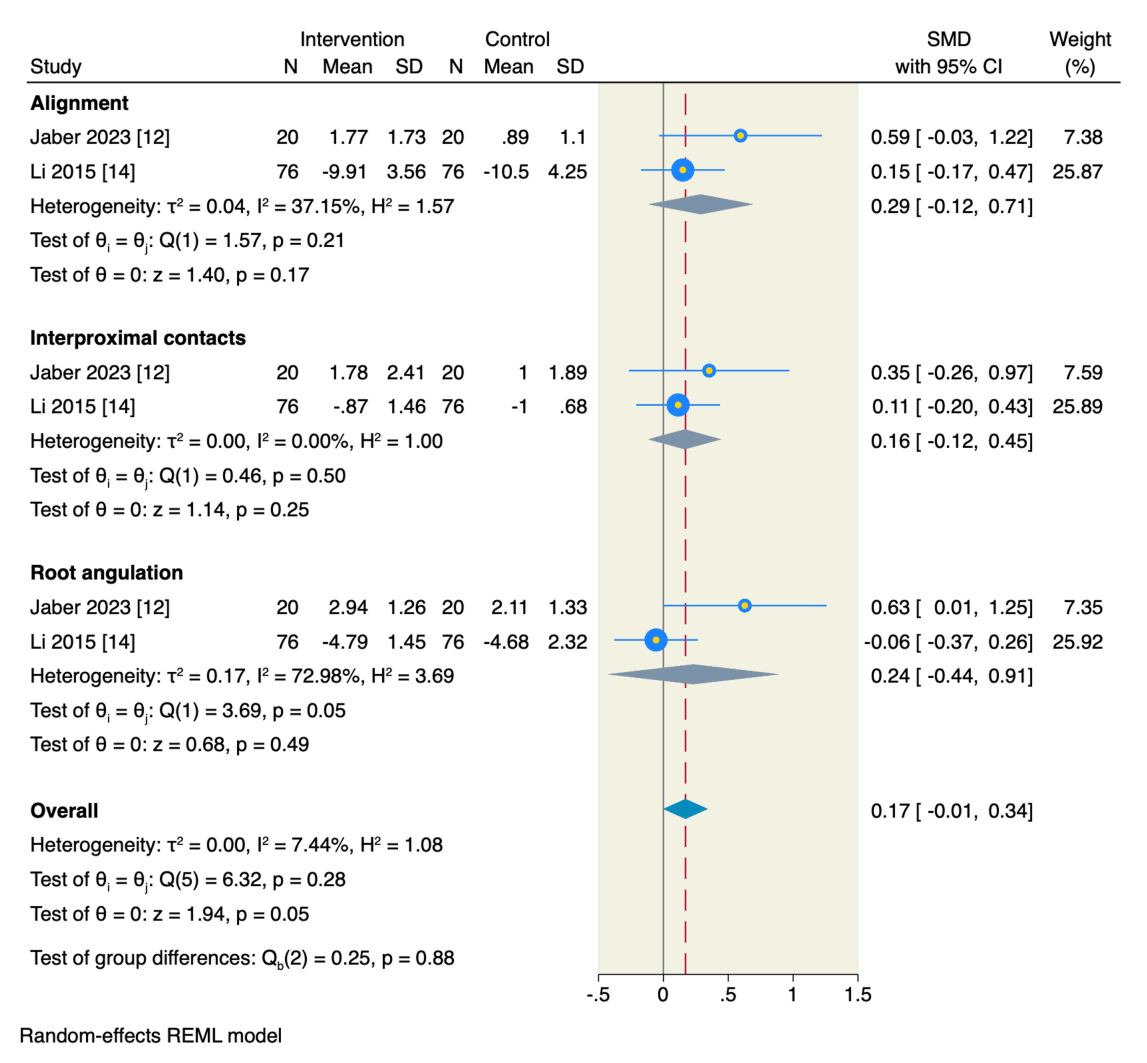
Random-effect model of the mean change of individual OGS score base domains. OGS: objective grading score [9] References: [12,14]

**Figure 7:**
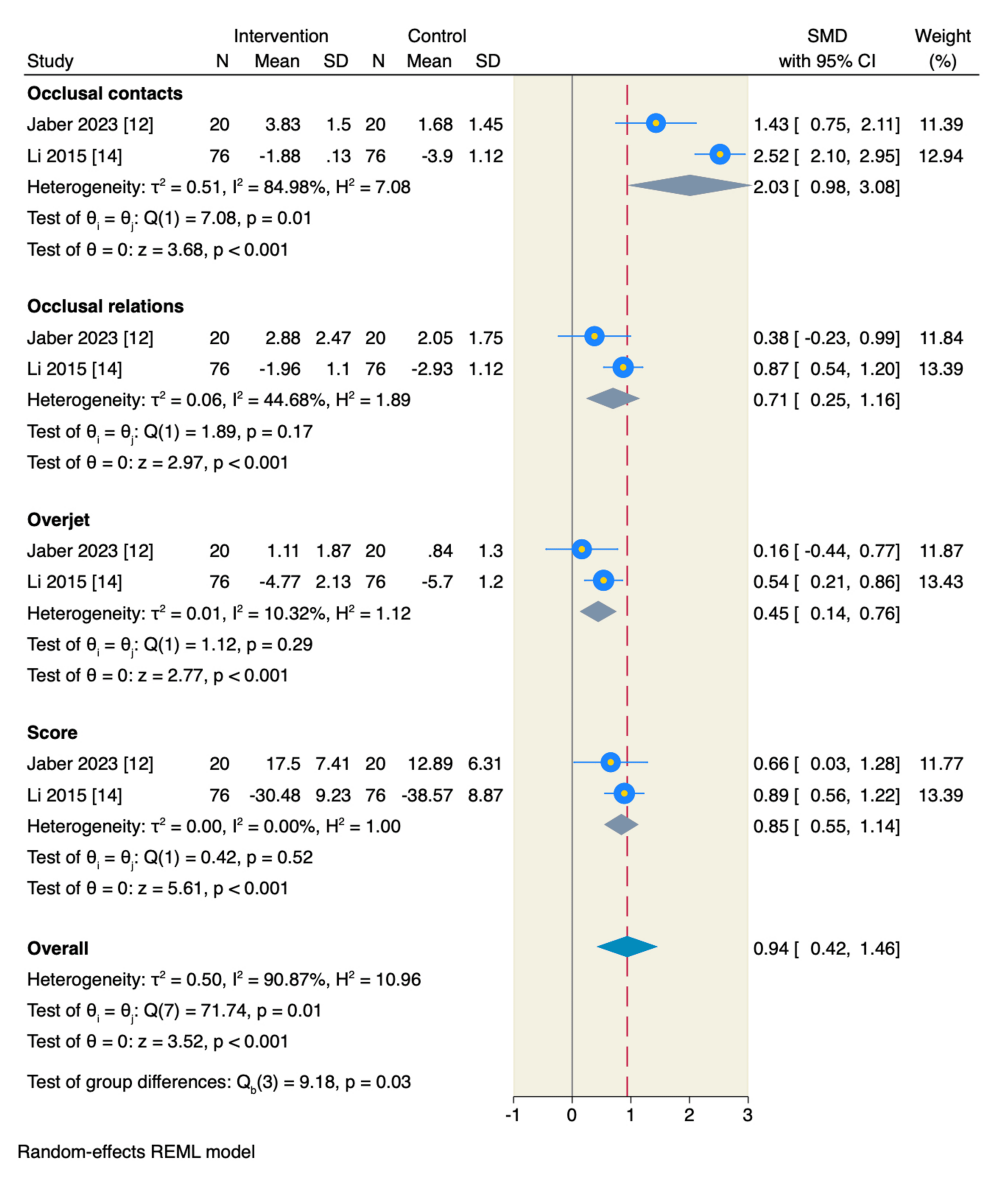
Random-effect model of the mean change of individual OGS score extended domains. OGS: objective grading score [9]. References: [12,14]

In addition, CA treatment resulted in better OGS scores in the following domains: occlusal contacts (SMD= 2.03, 95%CI: 0.98 to 3.08, p <0.001), occlusal relations (SMD= 0.71, 95%CI: 0.25 to 1.16, p <0.001), overjet (SMD= 0.45, 95%CI: 0.14 to 0.76, p <0.001), and overall score (SMD= 0.85, 95%CI: 0.55 to 1.14, p <0.001) (Figure 6). However, there was no significant difference between the two studied groups regarding the following OGS individual domains alignment (SMD= 0.29, 95%CI: -0.12 to 0.71, p =0.17), interproximal contacts (SMD= 0.16, 95%CI: -0.12 to 0.45, p =0.25), root angulation (SMD= 0.24, 95%CI: -0.44 to 0.91, p =0.49) (Figures 6, 7). 

Discussion 

The present systematic review and meta-analysis of seven RCTs and 402 patients is the most comprehensive study to date to compare CAs and FAs in malocclusion patients. Our study showed that patients treated with CA had better OGS scores in buccolingual inclination, marginal ridges, occlusal contacts, occlusal relations, and overjet, while there was no significant difference in other studied domains. Additionally, there were no significant differences in the other studied outcomes. 

Over recent years, CAs have been introduced as a popular orthodontic treatment option for patients with malocclusion [18]. Although there are still debates about the effectiveness of CAs in this context, the increase in QoL-related outcomes compared to conventional FAs has increased the interest in CAs [19]. QoL and health affect the psychological and social trust of individuals and are affected by the dentofacial issues caused by malocclusions [20]. A sub-division of QoL is oral-health-related QoL (OHRQoL), defined as the absence of psychological and physical problems related to oral health, and the related importance of self-confidence and psychological status that can affect the individual’s QoL [20]. Additionally, the anxiety and pain produced before and during different orthodontic interventions generally affect the OHRQoL [21]. The key determinant of continuing and not giving up the orthodontic treatment is the pain, as pain is considered an emotional status that individuals encounter during these types of surgical procedures, and reducing pain levels during and after these types of surgical interventions results in an increase in self-confidence and the willingness to continue treatments [21]. 

With regard to pain levels, it is seen that patients allocated to CAs showed no significant differences compared to those allocated to FAs. Despite the pooled result not being significant, individual studies showed heterogeneous results regarding pain levels. In the study done by Tunca et al. [16], they included 60 patients with mild or moderate malocclusion allocated to CA and FA groups. They reported that patients allocated to the CA group had significantly lower pain levels at the second and sixth hours of follow-up assessment, and at the third day of follow-up assessment as well. They reported that following the initial application of the arch wiring, edema and acute ischemia are commonly seen in the FA group and are often associated with increased pain levels. The same result was explained by another study, confirming that around 95% of the patients start to experience an increase in pain levels following the application of the arch wiring, while about 8% of the patients stop the treatment due to the increase in pain levels [22]. In other reports, the pain starts within the first 12 hours after arch wiring, peaks within the first day, and subsequently decreases after the third day of follow-up assessment [23]. 

In addition, reports by Fujimaya et al. [24] and Gao et al. [25] found that the pain levels peaked at the first day of assessment in both two studied groups and started to decrease afterwards; however, no significant differences were noted during the first few days of follow-up assessment. They also reported that there were significant results during the 14-day window of follow-up assessment between the studied groups, of which individuals treated with CAs experienced lower pain levels compared to those treated with conventional FAs. On the other hand, systematic reviews done by Cardoso et al. [26] and Mheissen et al. [27] found that the pain levels could vary between individuals with different malocclusion severity statuses, and this could bias the reported results among the included studies. In our study, we found that patients with mild or moderate malocclusion tend to have different pain scores compared to other severe forms. Additionally, studies paid attention to the tooth movement of the permanent teeth in patients treated with CAs, which could introduce a less painful sensation compared to those treated with FAs, all of which resulted in heterogeneous results [24,25]. 

Our review and meta-analysis showed that patients treated with CAs had no significant difference in terms of QoL scores compared to patients treated with FAs. In agreement with our findings, the study done by Tunca et al. [16] on 60 class one malocclusions found that only during the first day of follow-up assessment, the QoL scores, especially OHIP-14, were high in the CA group, while there was no significant difference concerning the results of OHRQoL-UK surveys between the two studied groups. These results are in agreement with findings by Antonio-Zancajo et al. [28] and Alfawal et al. [29], who reported that QoL measures were significantly higher in the CA group compared to conventional FA using OHIP-14 surveys; however, the type of malocclusion might have affected the reported results. On the other hand, Sharma et al. found no significant benefit of the CAs regarding QoL using the Child Oral Health Impact Profile-Short Form 19 (COHIP-SF 19) survey between the two studied groups [30]. Given the fact that the measurement of QoL is affected by many factors, the robust evidence of the current findings is uncertain. 

Additionally, when addressing the potential association between the orthodontic pain levels and related QoL measures, types of malocclusions and crowding levels should be taken into consideration [26]. Due to limited data available across the included studies, subgroup analysis based on different types of malocclusions of levels of crowding was not feasible. In addition, the biomechanics of the two interventions are different, of which the attachment used in conventional FAs might cause soft tissue damage, which in turn can increase pain levels of discomfort levels and subsequently lower the scores of QoL measures. Moreover, key factors that could have contributed to the observed individual results among the included studies were the number of CAs used, the duration of use, the types of attachments, and the order of tooth movement planning. CAs tend to have higher satisfaction scores among patients. 

Limitations 

Although our study is the first and most comprehensive study to date to assess the head-to-head comparison between CAs and FAs in malocclusion patients, there are some limitations to be taken into consideration. First, the small sample size across the included studies could limit the generalizability of the findings, and further studies with a large sample size and good power levels (90%) are needed to validate the current findings. Due to limited data available, we could not perform subgroup analysis based on malocclusion types, which could have introduced a degree of uncertainty, and further studies with different groups of arch length discrepancy with different types of attachments are needed to confirm and further validate the findings. Most of the studies were single-operator-based, in which the skilled operators might introduce different outcomes compared to non-skilled operators. 

## Conclusions

The present systematic review and meta-analysis of seven RCTs and 402 patients concluded that patients treated with CAs had better OGS scores, without significant differences in other studied outcomes compared to those treated with FAs. The current findings support the hype of the increased use of CAs in the orthodontic area; however, dentists should pay attention to the individualized assessment of each case. Further standard protocol-driven studies are needed to validate the current findings. 
